# Examining the Effects of Family and Acculturative Stress on Mexican American Parents’ Psychological Functioning as Predictors of Children’s Anxiety and Depression: The Mediating Role of Family Cohesion †

**DOI:** 10.3390/bs15081098

**Published:** 2025-08-13

**Authors:** Catherine Myshell Gonzalez-Detrés, Velma McBride Murry, Nancy A. Gonzales

**Affiliations:** 1 Department of Human & Organizational Development, Peabody College, Vanderbilt University, Nashville, TN 37235, USA; velma.m.murry@vanderbilt.edu; 2 Department of Health Policy, School of Medicine, Vanderbilt University, Nashville, TN 37235, USA; 3 Office of the Provost and Department of Psychology, Arizona State University, Tempe, AZ 85281, USA; nancy.gonzales@asu.edu

**Keywords:** acculturative stress, parenting, Family Stress Model, Mexican Americans, mental health

## Abstract

The combination of discrimination and cultural-contextual stressors associated with acculturation demands and immigration processes cause stressful conditions for Latinos above and beyond daily, stressful life events experienced in families. This in turn, can have repercussions on parent–child relationships and family dynamics. We hypothesized that acculturative and general family stress would be associated with increased parental depression, which would negatively affect family cohesion and parents, and that these disruptions would predict children’s internalizing symptoms. Accordingly, mothers and fathers (N = 467) completed questionnaires to describe their experiences of acculturative stress, with mothers also reporting on general family stress. Parent and children’s reports of parenting and family cohesion were also assessed. Structural equation modeling analyses were employed to examine the relationship between parental stress (acculturative and general family stress) and depression at Wave 1, with spillover effects on family cohesion, parenting, and children’s internalizing symptoms at Wave 2. Familial acculturative stress was positively associated with increased parental depression, compromised family relationships and parenting, and in turn, was linked to increased anxiety and depression in their children. In addition, family cohesion served a mediating role in families, helping to explain the pathway though which acculturative stress affects family relationships and children’s internalizing symptoms. This study addresses a critical gap in immigrant family adaptation research, highlighting the need for a cohesive model that integrates multiple stressors to capture their unique, collective, and cumulative effects.

## 1. Introduction

According to Mental Health America (2021), almost one-fifth of the Latino population, or approximately 10 million people, reported experiencing a mental health problem in the previous year. Several reasons have been offered to explain compromised mental health functioning of Latino children. Specifically, the combination of discrimination and cultural-contextual stressors associated with acculturation demands and immigration processes cause stressful conditions for this population above and beyond ubiquitous, daily, stressful life events experienced in families (McCord et al., 2019; White et al., 2009). Understanding the process by which cultural-contextual stressors are experienced by, and cascade through, immigrant families to affect parents and their children may offer insight on critical pathways to target in preventive interventions. Moreover, exploring these processes in Mexican American families is important, given that they are the largest ethnic and immigrant group in the United States, accounting for nearly 60% of the nation’s Hispanic/Latino population (Krogstad et al., 2023). Accordingly, informed by cultural stress theory and the family stress model, the current study examines the pathways by which acculturative stress and family stress differentially relate to Mexican American youths’ wellbeing (Conger et al., 1994; Salas-Wright & Schwartz, 2019). Special consideration will be given to exploring fathers’ and mothers’ unique experiences of acculturative stress on their parenting, and the mediating role of family cohesion in the face of parental stress.

## 2. Acculturative Stress and Depression in Mexican American Parents

Mexican American families face a disproportionate number of environmental stressors, such as poverty, neighborhood crime, and poor housing conditions that place them at greater risk for negative physical and mental health outcomes compared to their white counterparts (D’Anna-Hernandez et al., 2015; White et al., 2009). For example, studies have demonstrated that Latino children and families have been severely affected by immigration policies and rhetoric around their own criminalization, exacerbating negative mental, physical and emotional health outcomes (Ayón & Becerra, 2013). Such restrictive and targeted policies have not only constrained economic mobility and employment opportunities but have also threatened the safety and wellbeing of families, instilling great fear in the Latino community (Ayón et al., 2017; Roche et al., 2018; Wray-Lake et al., 2018). This fear has not been unique to those with undocumented statuses but has also impacted families with mixed statuses (undocumented, authorized, citizens) (Roche et al., 2018; Vargas et al., 2017).

Similarly, the anti-immigrant rhetoric employed by the president and the media has exacerbated attacks on the Latino population (Anbinder, 2019; Varela, 2019). In a report conducted in 2018, Lopez et al. (2020) found that one in four Latinos had experienced unfair treatment due to their background and language, often being insisted to return to their home country. Moreover, Almeida et al. (2016) found a positive relationship between anti-immigration policies at the state level and perceived discrimination of Latinos. The fear resulting from the uncertainty around new policies and future of immigration has led to greater isolation of Latinos to ensure safety for themselves and their families (Ayón & Becerra, 2013; Ayón et al., 2017; Cardoso et al., 2018; Salas et al., 2013). The infringement on parents’ safety and wellbeing has extended beyond themselves and altered the ways in which they interact with basic health services, their children’s school, and even their own families (Cardoso et al., 2018; Keller et al., 2010; Leidy et al., 2010).

In the current climate, Latino families must navigate these complex environmental stressors alongside the common challenges faced by all families. Major and daily life events, referred to here as family stress, arise from situations that while normative in nature can still be disruptive. These include experiences such as a family member having a medical crisis, separation from a loved one, or divorce. Scholars have categorized such experiences as general stressful life events (Bjørndal et al., 2024). These events have significantly contributed to symptoms of depression and anxiety (Bjørndal et al., 2024), and when compounded by other stressors, may further strain the family unit and compromise individual mental health functioning. Few studies, if any, have captured this.

In addition to these environmental and family stressors, Mexican American families are also tasked with negotiating different cultures and languages, also known as the acculturation process (Berry, 2008; Ward, 2006). Whereas the acculturation process is related to the undertaking of accommodating a new culture with an individual’s host culture, acculturative stress describes the extent to which families carry significant burdens associated with forcibly navigating different languages, cultures, and values, and as a function of being marginalized, often manifested as discrimination and prejudice (Gil et al., 1994; Vega et al., 1993). That is, families experience greater acculturative stress as they are increasingly forced to forego their native identity and practices (D’Anna-Hernandez et al., 2015). Therefore, acculturative stress is defined as the negative change in psychological well-being associated with the demands required of families functioning between two cultures (Berry, 1998; Lueck & Wilson, 2011).

Research studies examining acculturative stress have shown that chronic stress produced by acculturation is linked to greater evidence of depressive symptoms and compromised psychological and physical health, even above other cultural-contextual stressors (Calzada & Sales, 2019; Harris & Santos, 2020; Leon, 2014; Torres et al., 2012). For Mexican American families, acculturative stress plays an important role in parents’ experiences of psychological distress, as individuals who undergo chronic stress from acculturation are likely to suffer from anxiety and depression associated with that stress (Cervantes et al., 2019; Lozano et al., 2022). For example, one study found that when controlling for general perceived stress, acculturative stress was linked to higher levels of anxiety symptoms in Mexican American women compared to acculturation itself (Preciado & D’Anna-Hernandez, 2017). A similar impact is highlighted, though not as extensively, in fathers. In a systematic review of the important role of fathers in immigrant and refugee families, Bond (2019) found that fathers also experience acculturative stress due to changes in family roles, as they undergo a slower process of acculturation compared to their children, and subsequently face challenges associated with employment, social status, and poverty, which decrease their levels of self-esteem in leading the family. Low self-esteem and feelings of disappointment are then accompanied by greater levels of depression, which compromise parenting in both fathers and mothers (Ayón et al., 2017; Bond, 2019).

## 3. The Link Between Depression and Family Processes

Research has shown that general stress can impact parents’ emotional regulation which has implications for many aspects of family functioning, including parent–child relationships, family cohesion, and general lower life satisfaction (Cardoso et al., 2018; Lorenzo-Blanco et al., 2016). For mothers, specifically, studies have demonstrated that depression can significantly alter maternal sensitivity, such as the ability to appropriately perceive and respond to a child’s needs (Harris & Santos, 2020). Consequently, maternal psychological distress has been significantly linked to lower parent–child relationship quality (Murry et al., 2004), hostile (Deater-Deckard, 2004) and inconsistent parenting (Ayón et al., 2017). Both hostile and inconsistent parenting are thought to have detrimental effects on children, as the former reflects coercive and authoritarian parenting practices (Lovejoy et al., 2000), whereas the latter encompasses more variability in parental discipline and responsiveness (Ayón et al., 2017; Deater-Deckard, 2004). One study found that Latina mothers who experienced high levels of stress, were less likely to report involvement and less likely to display maternal positive verbalizations, which had indirect effects on their children’s internalizing symptoms (Figge et al., 2020).

Such processes can be exacerbated by additional forms of stressors, above and beyond those experienced in the family. For example, Camacho-Gonsalves (2002) found that Dominicans confronting multiple types of stressors, including discrimination, acculturative, and economic stress, were more likely to report greater depressive symptoms. Moreover, parents undergoing stress associated with discrimination, often found in conjunction with acculturative stress, have a harder time engaging in consistent discipline or warm and engaged parenting (Ayón et al., 2017; Deater-Deckard, 2004). This last component is critical because a fractured parent–child relationship can be a significant contributor to internalizing and externalizing behaviors in children (Manongdo & Ramirez Garcia, 2007).

Studies examining the pathway through which acculturative stress and parental depression affect parenting practices have identified family cohesion as an important mediator to buffer youth from these potentially negative family circumstances (Caplan, 2007). This predictive factor may be especially significant to consider in the Latino family unit, as Latino families are often characterized as a “system which helps provide support for its members against external stressors” (Hovey & King, 1996, p. 27). Family cohesion, defined as “the emotional bonding that family members have toward one another” (Olson et al., 1979, p. 70, as cited in Marsiglia et al., 2009) has been found to protect against risky behaviors such as substance use and delinquency (Coohey, 2001, as cited in Marsiglia et al., 2009). Further, a previous study examining the impact of familism on Mexican American children’s internalizing symptoms found that family cohesion served as mediator in predicting that relationship (White & Roosa, 2012). The social support garnered from the family increases the parents’ ability to remain resilient and engaged in challenging times (Cabrera et al., 2021), suggesting that family cohesion could explain the linkage between parental depression and parenting practices produced as a result of experiencing acculturative stress (Behnke et al., 2008). Though the studies cited are representative of the role of family cohesion in buffering adolescents from negative outcomes, we suggest similar patterns may exist for children in middle childhood.

## 4. Anxiety and Depression in Latino Children

A plethora of studies have demonstrated strong associations between parenting practices and development of internalizing behaviors in early childhood and adolescence (i.e., anxiety, depression; Calzada et al., 2017; McLeod et al., 2007). In Mexican-origin families, authoritarian parenting (low responsiveness, high demandingness) predicted anxiety and depression in early childhood, with stronger links found between authoritarian parenting and depression than with anxiety (Calzada et al., 2017). Another study exploring parental acceptance-rejection found that rejection, as indicated by hostility, indifference, and lack of affection, was significantly associated with higher scores of internalizing behaviors on the Child Behavior Checklist (Rothenberg et al., 2022). During adolescence, these findings persist such that Mexican-origin youth experience increases in symptomatology as a result of harsh parenting (White et al., 2015). On the contrary, parental warmth and consistent discipline have served as protective factors against negative developmental outcomes in children, with studies showing that Latino parents create nurturing and engaging environments that produce this type of parenting (Cabrera et al., 2021; White et al., 2015). Consequently, examining parenting processes are critical to understanding how Mexican American children are able to cope with stressful events in their environment and avert internalizing behaviors. While these pathways have been well documented in relevance to general stressful life events, less is known about how this process unfolds as a consequence of acculturative stress.

## 5. The Present Study

**Figure 1:**
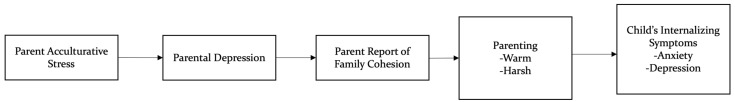
Hypothesized model for mothers and fathers.

The present study is informed by cultural stress theory which posits that stressors specific to new cultural immersion impact many first- and second-generation immigrants (Salas-Wright et al., 2021). A key characteristic of the cultural stress theory is the accumulated effect of many stressors that have implications for family functioning and youth’s behavioral and psychological health outcomes (McCord et al., 2019; Salas-Wright et al., 2021). For example, youth’s perception of discrimination in 9th grade has been linked to heightened depression in 11th grade (Basáñez et al., 2013), and acculturation-specific conflict between parents and children was also associated with depressive symptoms in youth during early and middle adolescence (Bámaca-Colbert et al., 2012). Though it is clear that cultural stressors experienced by youth are related to negative developmental trajectories, only a few studies have examined how parents’ cultural stressors have the same implications for youth, through family processes (Lorenzo-Blanco et al., 2016). The Family Stress Model (FSM) substantiates this pathway, analyzing how parents’ perceptions of acculturative stress impact family functioning and are related to children’s psychological health outcomes (Conger et al., 1994). Though the impact of acculturative stress in Mexican American families has been previously studied (Calzada et al., 2019; White et al., 2009), researchers have focused primarily on Latino children and youth, with limited attention given to examining how acculturative stress affects parents, and even less attention given to including both mothers and fathers (Calzada et al., 2017; Lorenzo-Blanco et al., 2016). In their review of studies examining parental acculturative stress, Miller and Csizmadia (2022) found that only four studies had looked at both mothers and fathers’ reports of acculturative stress, and only two of those studies linked it to child outcomes. None of these studies, however, had investigated family cohesion as a determining factor in these relationships. To begin to fill this void, a secondary data analysis of Mexican American parents and children was conducted to address the following questions through the hypothesized model (see Figure 1):

- Question 1 (Q1): What is the relationship between parents’ acculturative stress and depression, and subsequently, their reports of family cohesion? What is the role of family cohesion in predicting parenting behaviors and subsequently, children’s internalizing behaviors?
- Question 2 (Q2): What role does family cohesion play in mediating the indirect impact of parental acculturative stress on children’s psychological health?
- Question 3 (Q3): What role does family stress play in predicting the relationships between predictor and dependent variables?

## 6. Methods

Data from an ongoing longitudinal study examining culture and context in Mexican American families was utilized for this secondary data analysis. The study was conducted in compliance with the respective Internal Review Board, and APA ethical standards. Further details on study participants, recruitment, or procedures can be found in Roosa et al. (2008).

### 6.1. Participants

For eligibility, participating families had to meet five criteria at Time 1: (a) they had to have a 5th grader attending one of the sampled schools; (b) the mother was the child’s biological parent, lived with the child, and self-identified as Mexican or Mexican American; (c) the child’s biological father self-identified as Mexican or Mexican American; (d) the target child did not suffer from any learning disabilities; and e) the child was not living with step-father or mother’s boyfriend. In total, 750 families (mother–child dyads) participated at Time 1 (T1).

Of the 750 families at T1, 22.9% were single-parent families and 77.1% were two-parent families. Although participation for fathers was optional, 467 (81.9%) fathers of the 570 two-parent households were also interviewed, demarcating the final sample for the current study (N = 467). In comparison to previous research with Mexican American families, the sample in this study was socioeconomically and linguistically diverse (Roosa et al., 2008), with the range of family income falling between US$5000 to US$95,000. The sample was intentionally recruited from various communities to include participants that represented Mexican Americans across levels of income, generation status, acculturation level, and cultural experiences, following an ethnic homogenous design, which was relevant to the purpose of the project (Cauce et al., 1998; Roosa et al., 2008).

The mean age of the youth was 10.42 (SD = 0.55), with females representing less than half of the sample (48.7%). Almost 75% of mothers and 80% of fathers were born in Mexico, and about 70% of adolescents were born in the United States; 30.2% of mothers, 23.2% of fathers, and 82.5% of youth were interviewed in English. At Time 2 (T2), 711 of the 750 families were reinterviewed, resulting in a 95% retention rate. There were no significant differences between participating families and participants lost (see Roosa et al., 2008). For the current study, only data with two-parent households (N = 467) was utilized, given the nature of the analyses.

### 6.2. Procedure

Children were recruited from 47 public, religious, and charter schools, with more than 20 Latino students in the fifth grade and with rich cultural contexts (see Roosa et al., 2008 for more information). Schools were identified through a combination of random and purposive sampling to represent the diversity of Mexican American families across the city. The adolescents in these schools were given recruitment materials to share with their parents in order to indicate their willingness to participate. Mothers and children were required to participate together, and fathers’ participation was optional. Once families agreed to participate, data collectors scheduled In-home Computer Assisted Personal Interviews, which allows for families to have questions and answers read to them in hopes of reducing variation in the data associated with literacy. Interviews lasted about 2.5 h, with each member being interviewed separately by bilingual interviewers to promote privacy. Interviewers received around 40 h of training, which covered project goals, information on the target population, and interviewing strategies in the home. During the interviews, data collectors introduced themselves, reviewed consent/assent forms and answered any questions regarding the Certificate of Confidentiality and study procedures. Each participating family member received US$45 at T1 and US$50 at T2.

### 6.3. Instruments

#### 6.3.1. General Family Stress

To capture family stress at T1, the Multicultural Events Scale for Adolescents (MESA; Gonzales et al., 2001) family trouble/change subscale was adapted for the mother, the primary caregiver examined in this project. Mothers reported on 8 items describing if they experienced events such as a close family member dying or moving far away from family or friends, with 1 signifying the event happened, and 2 signifying the event did not occur, *α* = 0.46. The lower reliability may be explained by the small number of items, or the complexity of the construct given the range of situations and events that can be stressful for families (Schmitt, 1996). Scale responses were recoded from 2 to 0 and summed for the total count of stressful events.

#### 6.3.2. Acculturative Stress

Reports of acculturative stress for mothers and fathers were assessed at T1, using the English Competency Pressures subscale of the Multidimensional Acculturative Stress Inventory (MASI; Rodriguez et al., 2022). Parents responded to 8 items corresponding to the extent to which they experienced difficulties speaking English or people treated them rudely or unfairly because they did not speak English well on a Likert scale ranging from (1) not at all true to (5) very true. Alpha level was 0.87 for mothers and 0.83 for fathers.

#### 6.3.3. Parental Psychological Functioning

Mother’s and father’s experience with depression was captured through the Center for Epidemiologic Studies Depression Scale (CESD-D; Radloff, 1977). This scale consists of a self-report questionnaire with 20 items related to an adult’s depressed mood, feeling of helplessness and hopelessness, sleep disturbance, and others. Responses were measured on a Likert scale ranging from (0) rarely or none of the time (less than one day) to (3) most or all of the time (5–7 days). Some items were reverse coded to ensure higher scores indicate greater levels of depression (e.g., “You enjoyed life.”). Alpha for mothers was 0.77 and for fathers, 0.80.

#### 6.3.4. Family Cohesion

To assess family cohesion, or the emotional bonding between family members (Olson et al., 1983), the cohesion subscale of the Family Adaptability and Cohesion Evaluation Scales II (FACES II) was used. This measure includes items to measure the extent to which “family members are supportive of each other during difficult times” or “family members feel very close to each other.” The Likert scale for these items ranged from (1) almost never or never to (5) almost always or always. Both mothers and fathers responded to these items at T2, *α* = 0.83.

#### 6.3.5. Parenting Processes

Children’s reports and parents’ self-report of warm (8 items) and harsh (8 items) parenting was measured using the Children’s Report of Parental Behavior Inventory (CRPBI; Schaefer, 1965) at T2. Participants indicated the extent to which the mother and/or father “made [them] feel better after talking over [their] worries with her/him” or “screamed at [them] when [they] did something wrong”. Items were scored on a Likert scale ranging from (1) almost never or never to (5) almost always or always. Across reporters and parenting behaviors, alpha levels were between 0.70 and 0.85. This scale has been tested and validated with a Hispanic sample (Knight et al., 1994).

#### 6.3.6. Youth’s Internalizing Symptoms

The Diagnostic Interview Schedule for Children Version IV (Shaffer et al., 2000) assessed psychiatric disorders and symptomatology in children. The C-DISC, as it is also called, has been successfully translated to Spanish from psychometric work performed in Puerto Rico (Bravo et al., 1993). The C-DISC captures diagnosis variables, symptom counts, and criteria counts for each of the disorders. Anxiety and depression symptom counts were summed to represent internalizing symptoms at T2, *α* = 0.67.

### 6.4. Analytic Plan

A correlational analysis in IBM SPSS Statistics (Version 30) was utilized to explore the various relationships between the variables examined in this study. The correlation tables (Table 1 and Table 2) include: (1) parents’ reports of their experiences of stress, psychological functioning, and family processes; (2) parents’ and children’s reports of parenting strategies; and (3) children’s reports of their internalizing behaviors. Thereafter, structural equation modeling (SEM) was employed to identify pathways through which acculturative stress at T1 predicted family functioning and in turn children’s internalizing problems at T2 (Q1). SEM also demonstrates the role of family cohesion across the pathway from parental acculturative stress at T1 to youth’s reports of anxiety and depressive symptoms at T2 (Q2). For the current study sample size, SEM was found suitable as previous research indicates that at minimum, the sample size should be of 100 or 200 to conduct this type of analysis (Wolf et al., 2013). A priori power analyses were conducted using RMSEA-based noncentral chi-square methods to assess the adequacy of the sample size for structural equation modeling (SEM). The hypothesized model (Figure 1) had 9 degrees of freedom and was tested separately across two subsamples of total, N = 475. Using standard thresholds for model fit (RMSEA = 0.05 for close fit versus RMSEA = 0.08 for poor fit) and α = 0.05, the estimated statistical power for each subsample was approximately 0.92. This exceeds the conventional 0.80 threshold, indicating that each model had sufficient power to detect poor-fitting models, thereby supporting the stability and reliability of the SEM results. Assumptions were met for the path analyses, as no evidence of multicollinearity was found based on bivariate correlations between predictors, all of which were below the recommended threshold of 0.80 (Kline, 2016). Further, Skewness and Kurtosis coefficients demonstrated normality in the data, as all fell between −2 and +2. Only one variable had a kurtosis coefficient of 2.13 which is still acceptable given the sample size (Kline, 2016).

Finally, analyses were conducted to examine the unique and combined contributions of two sources of stress (e.g., general family stress and acculturative stress; Q3). For missing data, maximum likelihood estimation procedures were applied using the lavaan package in R, which attends to biased parameter estimates (Arbuckle & Wothke, 1999). The models’ goodness of fit was assessed through various indicators including, chi-square, root mean square error of approximation (RMSEA), standardized root mean square residual (SRMR), and the comparative fit index (CFI). An acceptable model fit should show an RMSEA value closer to 0 (less than 0.05), a SRMR value less than 0.05 or 0.08, and a CFI value greater than 0.90 (Hu & Bentler, 1999).

## 7. Results

### 7.1. Correlational Analyses

Correlational analyses showed that parents’ reports of experiencing acculturative stress were positively associated with depression, with slightly stronger reports from mothers (Table 1) compared to fathers (Table 2), (*r* = 0.28, *p* < 0.01) and (*r* = 0.27, *p* < 0.01), respectively. Family stress was also associated with increased reports of depression among mothers (*r* = 0.12, *p* < 0.01). Both mothers’ (*r* = −0.30, *p* < 0.01) and fathers’ (*r* = −0.35, *p* < 0.01) experiences of depression were associated with a decrease in family cohesion, reflecting less evidence of emotional support and bonding in families. Family cohesion was positively associated with children’s reports of receiving warmth and supportive parenting (*r* = 0.17, *p* < 0.01) and less likely to experience harsh parenting (*r* = −0.11, *p* < 0.05) from their mothers. Similar trends were present for fathers, as examination of children’s reports revealed that family cohesion was associated with both warm parenting (*r* = 0.09, *p* > 0.05) and harsh parenting (*r* = −0.13, *p* < 0.05), with slightly stronger associations for harsh parenting. Based on the child’s reports, mother’s warm (*r* = −0.20, *p* < 0.01) and harsh parenting (*r* = 0.24, *p* < 0.01) were significantly associated with the child’s internalizing symptoms, in the expected direction. Finally, children’s reports of the association of their fathers’ parenting behavior to symptoms of internalizing behaviors, were also in the expected direction, specifically, warm parenting (*r* = −0.21, *p* < 0.01) and harsh parenting (*r* = 0.28, *p* < 0.01). Overall, these results demonstrate low to moderate significant associations among the selected study variables suggesting their utility for testing the research questions (Akoglu, 2018).

### 7.2. Measurement Models

#### 7.2.1. Mothers’ Model

To assess best model fit, measurement models were analyzed for mothers’, fathers’, and children’s reports of warm and harsh parenting in relationship to acculturative stress. The measurement model drawing from mothers’ reports of their own parenting demonstrated a poor fit, χ2 (14, N = 467) = 49.49, *p* = 0.000; RMSEA = 0.07, SRMR = 0.06, and CFI = 0.83, all loadings, except for parenting to internalizing behaviors, <0.001. When combining children’s and mother’s reports of mother’s parenting together, the model also demonstrated a poor fit, χ2 (27, N = 467) = 102.46, *p* = 0.000; RMSEA = 0.08, SRMR = 0.06, and CFI = 0.75, all loadings < 0.05. In contrast, the measurement model for only children’s reports of mother’s parenting demonstrated a good fit, χ2 (9, N = 467) = 10.28, *p* = 0.33; RMSEA = 0.02, SRMR = 0.03, and CFI = 0.99, all loadings < 0.001. Moreover, the parenting construct with children’s reports demonstrated high loadings for both warm parenting (loading = 0.46, *p* < 0.001) and harsh parenting (loading = −0.50, *p* < 0.001). Accordingly, only children’s reports were considered for the measurement model depicting acculturative and family stress, which demonstrated excellent fit, χ2 (13, N = 467) = 15.11, *p* = 0.30; RMSEA = 0.02, SRMR = 0.03, and CFI = 0.99, all loadings < 0.05.

#### 7.2.2. Fathers’ Model

Factor loadings for fathers’ parenting were also analyzed, with fathers’ reports, child’s reports, and combined reports from fathers and children. Similarly to the mother’s model, utilizing fathers’ reports of their own parenting did not demonstrate a good fit, χ2 (9, N = 467) = 38.09, *p* = 0.000; RMSEA = 0.08, SRMR = 0.07, and CFI = 0.89, most loadings < 0.05. Furthermore, children and fathers’ combined reports also did not make for an adequate model fit, χ2 (20, N = 467) = 130.18, *p* = 0.000; RMSEA = 0.11, SRMR = 0.09, and CFI = 0.70, all loadings < 0.05. However, the children’s report of fathers’ warm and harsh parenting demonstrated the best fit, χ2 (9, N = 467) = 6.89, *p* = 0.65; RMSEA = 0.00, SRMR = 0.03, and CFI = 1.0, all loadings < 0.05. The non-significant chi square in the models with children’s reports of parenting demonstrate that there is low discrepancy between the sample and the fitted covariance matrices. That is, compared to mothers’ and fathers’ reports of their own parenting, children’s reports are more conducive to explaining how parenting, compromised by experiencing stress, can impact children’s psychological wellbeing.

#### 7.2.3. Full Structural Models

**Figure 2:**
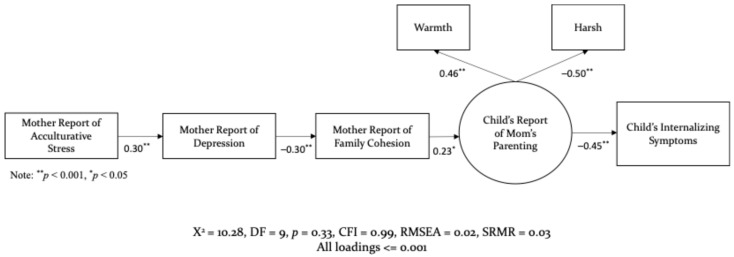
Structural Model for Mothers.

**Figure 3:**
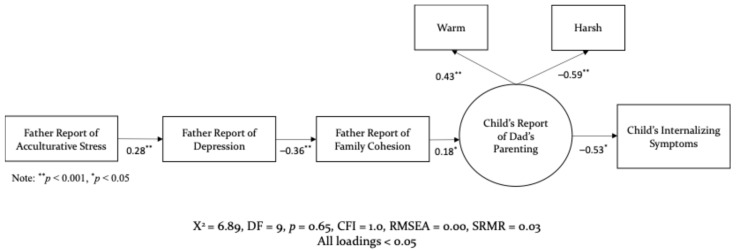
Structural Model for Fathers.

The first research question focused on examining the pathways through which acculturative stress affected parenting, through family processes, and in turn predicted a child’s mental health outcomes. The results for mothers’ and father’s structural models are illustrated in Figure 2 and Figure 3. As hypothesized, maternal reports of acculturative stress were directly associated with heightened maternal depression (*β* = 0.30, *p* < 0.001), which in turn was negatively associated with family cohesion (*β* = −0.30, *p* < 0.001). These represent moderate effect sizes, indicating meaningful associations between culturally specific stressors, maternal mental health, and family functioning. A similar pattern emerged for fathers such that experiencing greater acculturative stress was associated with increased depression (*β* = 0.28, *p* < 0.001), and subsequently, a decline in family cohesion (*β* = −0.36, *p* < 0.001). These findings also reflect moderate to moderately strong effects. Although the overall pathway structure was similar for both parents, the strength of associations differed. Mothers experienced slightly greater depressive symptoms in response to acculturative stress, while fathers’ depressive symptoms were more strongly associated with diminished family cohesion. These findings suggest gender-specific patterns in how stress is internalized and expressed in the family context.

The second research question examined the mediating role of family cohesion. As hypothesized, greater family cohesion was significantly associated with children’s reports of warm and supportive maternal parenting (*β* = 0.23, *p* < 0.05), representing a small to moderate effect. In turn, youths’ positive perceptions of maternal parenting were negatively associated with internalizing symptoms, such as anxiety and depression (*β* = −0.45, *p* < 0.05), reflecting a moderate to large effect. A similar pattern emerged for fathers: higher family cohesion was positively associated with supportive paternal parenting (*β* = 0.18, *p* < 0.05), a small effect, which subsequently predicted lower levels of youth internalizing symptoms (*β* = −0.53, *p* < 0.05), indicating a large effect. These findings underscore the important function of family cohesion amid acculturative stress. Specifically, family cohesion explained the impact of stress on parenting in two key ways—by reducing the use of harsh parenting behaviors and by promoting warm, supportive practices, even in the presence of external stressors.

**Figure 4:**
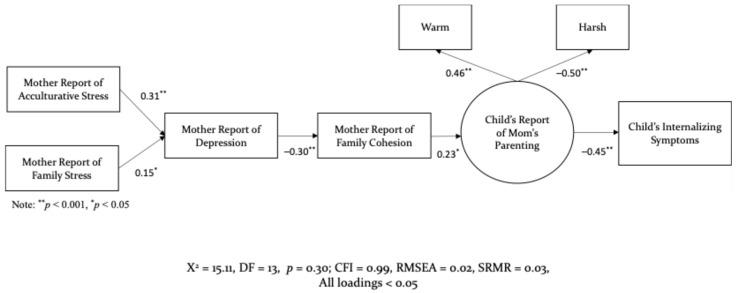
Structural Model for Mother with Family Stress.

The third model examined whether different sources of stress—namely acculturative stress versus general family stress—exerted distinct direct and indirect effects on family processes that influence children’s mental health. As illustrated in Figure 4, the path from acculturative stress to maternal depression was stronger (*β* = 0.31, *p* < 0.001) than the path from general family stress to maternal depression (*β* = 0.15, *p* < 0.05). The association between acculturative stress and depression reflects a moderate effect, while the path from general family stress reflects a small effect. This pattern highlights the unique and more substantial contribution of acculturative stress to Latina mothers’ psychological wellbeing. Although similar effects may be present among fathers, comparable data were not collected at that time point; therefore, a parallel model could not be tested for fathers.

Taken together, these findings demonstrate that acculturative stress exerts a significant and distinct influence on parental well-being and parenting behavior over time, with family cohesion serving as a critical intervening factor that promotes more adaptive parenting and, in turn, better mental health outcomes for youth. These results highlight important pathways through which culturally specific stressors shape family dynamics and point to modifiable targets for future intervention. The implications of these findings are explored in the discussion that follows.

## 8. Discussion

Consistent with previous work on the impact of stress on parenting, the current study supports the theory that stress diminishes the quality of parenting, and that this has implications for child adjustment (Conger et al., 1994). The results demonstrate that acculturative stress leads to heightened depression in parents, compromised family relationships (e.g., family cohesion) and parenting practices, that in turn, elevates risk for anxiety and depression in children. The scope of the original family stress model was designed to inform studies to examine ways in which general stressful life events, such as loss of job, family death, divorce and family affect the family unit through economic pressure, as well as disruptions in family functioning, which was conjectured to affect parenting practices. While a plethora of studies have evolved from this theory, making significant contributions to the field of family stress research, more studies examining cultural-contextual stressors are needed. For example, this theory is void of specific attention to the potential implications of perceived discrimination or acculturation as major stressors for racial or ethnic groups (Conger et al., 1994; Murry et al., 2018). As a complement to the FSM, cultural stress theory allows for the inclusion of stressors that may directly emerge from adjustment and adaptation associated with immigration, and predict youth outcomes pertaining to behavioral and psychological health (McCord et al., 2019; Salas-Wright & Schwartz, 2019) Admittedly, to our knowledge, none have examined the unique and cumulative effects of general stress life events and acculturative stress effects on families, in particular, Mexican American parents and their children (Miller & Csizmadia, 2022). The current study sought to fill this void.

### 8.1. Mothers and Fathers Pathways (Q1)

The first aim of the study was to consider how acculturative stress is driven both by parents’ appraisal of these stressors and parents’ reaction to the demands and expectations to acculturate into the dominant society (Berry & Annis, 1974). The underlying assumption of this aim is that acculturative stress requires a unique set of coping strategies and in turn may evoke different effects on parental psychological functioning, namely elevating depression. Specifically, the current study found that the positive relationship between acculturative stress and depression was stronger for mothers, compared to fathers. This finding is consistent with literature examining the role of mothers as the primary caregivers and their vulnerability to greater stressors in schools, healthcare systems, and neighborhoods, that could place them at greater risk of being discriminated against (Caplan, 2007; Calzada et al., 2019). This is especially relevant given the hostile political discourse that has led to greater discrimination and prejudice against Latino groups. Preliminary analyses in a recent study conducted by UC San Diego found that mothers reported greater anxiety and depression as a result of heightened discrimination due to anti-immigrant rhetoric and treatment (Kiderra, 2022). Further, language barriers, a key source of acculturative stress, may generate greater difficulties for mothers in finding networks of support or even resources or services that help them navigate the parenting demands that are required of primary caregivers, especially when attempting to maintain key cultural values amidst navigating cultural stressors (McWhirter & Donovick, 2022).

In the current study, the inverse relationship between depression and family cohesion was stronger for fathers than mothers. A potential explanation for this may be mothers’ and fathers’ differential reactions when experiencing stress, where fathers are more likely to externally portray their response to stress, compared to mothers who internalize those emotions (Shafer et al., 2017). That is, mothers may exhibit more depressive symptoms, whereas fathers may externalize their frustrations through actions that fracture the family unit, namely family cohesion. Previous studies have supported this hypothesis by concluding that parent–child connections are more vulnerable to negative changes due to fathers undergoing stress and depression, than mothers (Cummings et al., 2004). The differences in these reactions may derive from gendered parenting practices prevalent in immigrant communities where mothers are expected to manage the majority of household and caregiving responsibilities (Galanti, 2003). With mothers having a more active role in their child’s life, they are exposed at greater rates to other sources of stress that implicate not only their wellbeing, but their child’s as well. Challenges, such as dealing with stressors in schools or healthcare institutions in addition to the home, may place mothers at a greater risk of depleting their reserve capacity, thereby making them more vulnerable to poor mental health outcomes (Bennett, 2020). Alternatively, cultural norms such as *machismo* may lead fathers to underreport psychological distress or experiences of stress. Future assessments of parental psychological distress may benefit from observational measures or reports from multiple informants. Further, future researchers should target educational interventions that inform mothers and fathers alike of the impact of gendered roles on the physical and psychological reserve capacity of each member of the family. This can promote greater gender equity in parenting practices, reducing the disproportionate stress faced by mothers. Practitioners should also be cognizant of these gendered roles and, when possible, offer educational material or programs that encourage and equip mothers and fathers alike to share caregiving responsibilities for the benefit of the family unit. These efforts can enhance the quality of life and health of Latino parents by protecting them from being overburdened by additional stressors than those faced in their daily life.

### 8.2. The Mediating Role of Family Cohesion (Q2)

The current study also elevates the importance of explanatory factors as mechanisms that can potentially mitigate the negative effects of both acculturative and general stressful life events on families. As an important cultural value in Latino families, emotional bonding or family cohesion can provide important protection as parents navigate the effects of acculturative stress. That is, results offer support for the mediating nature of family cohesion in predicting the use of warmth parenting despite the demands and challenges emerging from stressful life events. Specifically, although exposed to acculturative stress, parents were more likely to express warm parenting and less likely to exhibit harsh parenting practices. This finding offers support for the important role of familism or *familismo* in Latino groups (Lorenzo-Blanco et al., 2016). A more recent study, by Miller and Csizmadia (2022) offers insight that may be useful to glean reasons for inclusivity of family cohesion as a buffering mechanism between parental acculturative stress and youth’s psychological health outcomes. Specifically highlighted in this study is the conclusion that the directionality of the relationship between acculturative stress and family cohesion has not yet been extensively explored (Miller & Csizmadia, 2022). In fact, the current study is the first to test pathways by which family cohesion may serve as a mediating factor by which acculturative stress impacts parent–child relationships and children’s anxiety and depression.

Recognizing family cohesion as a mediating mechanism is especially meaningful given that it is a quality that can be intentionally nurtured through various interventions. For example, schools can create opportunities for families to strengthen communication and mutual support through shared goals, such as promoting children’s academic success. This can be accomplished by implementing culturally responsive practices that affirm families’ cultural values and ways of being, thereby enhancing parents’ sense of belonging. Further, organizations or practitioners interested in working with Latino families can enhance parental self-efficacy by equipping them with tools to openly communicate with their children about their acculturation experience. These efforts can encourage greater dialog in the family, and subsequently, greater closeness among members.

### 8.3. The Role of General Family Stress (Q3)

This study also aimed to extend extant studies of acculturative stress beyond focusing on the mediational pathways of socioeconomic status and parental depression (Emmen et al., 2013) or as a single item included in general family stress models (Lorenzo-Blanco et al., 2016) by examining its contribution in conjunction with a measure of general family stress. The impetus for this approach was guided by two studies that found that acculturative stress was more predictive of maternal depression and anxiety than general perceived stress or perceived discrimination (D’Anna-Hernandez et al., 2015; Preciado & D’Anna-Hernandez, 2017); and, a previous study using this same data, that explored the pathways from three contextual stressors (i.e., financial hardship, neighborhood danger, and acculturative stress) to parental depression to warm and harsh parenting (White et al., 2009). In all, these previous studies demonstrated the need for incorporating multiple stressors to differentiate the impact each could have on parental wellbeing. Accordingly, findings from the current study demonstrate that acculturative stress may be evincing a greater burden on parents, as it was a stronger predictor of depression than association between general family stress and parental psychological functioning. These findings suggest the need to recognize the unique effects of cultural stressors on immigrant families. In this instance, acculturative stress for Latino families creates demands and challenges that go above and beyond general stressful live events experienced in non-immigrant families. Of importance is the dual and cumulative effect of both sources of stress on families that may overload the demands and capabilities of families, with spillover negative consequences to address needs of family members. Undoubtedly, immigrant families must learn how to navigate stressors emerging from transitioning to a new cultural context in addition to facing normative stressors. Understanding this phenomenon and intervening at appropriate windows of development, by fortifying parents’ coping strategies, could prevent maladaptive parenting practices and reduce risks for negative psychological health outcomes in Latino youth.

## 9. Implications

The findings from this study have important practical and theoretical implications. First, practitioners should be aware that when serving Latino populations, it is imperative to consider the ways in which the acculturation process inflicts added stress on parents, given that it is the most addressed topic when it comes to Latinas’ maternal mental health (Preciado & D’Anna-Hernandez, 2017). To address this, culturally tailored parenting programs could target issues of immigration, trauma, and stress by creating spaces where parents can openly discuss the challenges they have experienced while adapting to a new country. Delivering these in a group format may also foster greater participation from fathers, particularly if they observe other men sharing similar experiences.

Practitioners should be cognizant of how stress may manifest itself internally or externally in parents and subsequently, how it could negatively impact family functioning. Inversely, family-based interventions could be implemented to alleviate tension and stress buildup in the family unit in an effort to promote both family processes and members’ psychological functioning. These interventions can focus on promoting discussions around intergenerational acculturation gaps, increasing mental health awareness, strengthening family routines, and fostering psychosocial coping skills in parents and children alike. Addressing this in families with high levels of acculturative or family stress early on, can have productive consequences for averting depression and anxiety in Latino children. Finally, we call on other researchers to examine similar processes in other Latino populations, since this only speaks to the experiences of Mexican American families and could manifest itself differently between groups.

Another noteworthy finding highlights the importance of including multiple reporters in families lived experiences, for doing so will produce a more robust account of how these experiences are impacting the family through the perspectives of more than a single family member. Most studies investigate the impact of acculturative stress on families by relying on mothers as the primary reporter, noting that mothers are the primary family caregivers (Calzada et al., 2019; Miller & Csizmadia, 2022; Zeiders et al., 2015). Consequently, few studies have included both parents, and even less have reports from Mexican American mothers and fathers in studies of acculturative stress and its impact on their children (Miller & Csizmadia, 2022; White et al., 2009). The current study expands research design approaches to studying everyday life experiences of Mexican American families and revealed unique findings for mothers and fathers. For example, while intuitively, all family members should be affected by acculturative stress. Empirical testing is needed to corroborate this conjecture. In addition to providing parent reports, the inclusion of child reports on parenting is important to highlight as it describes their lived experiences of receiving warm or harsh parenting rather than depicting parents’ intentions which may be less representative of what actually occurs (Van Roy et al., 2010). This is consistent with previous studies that have found children’s perceptions to be more reflective of outcomes such as internalizing or externalizing symptoms (De Los Reyes & Kazdin, 2005).

Theoretically, the current study illuminates the need for a cohesive model that incorporates both the personal and familial contribution of cultural stress in reference to youth outcomes. Family stress research has traditionally examined pathways by which negative stressful life events affect parenting and family processes, with a lack of focus on cultural stressors. Alternatively, cultural stress theory has begun to incorporate family processes in the link between cultural stress and maladaptive outcomes in immigrants, with restricted knowledge of how those pathways unfold for parents’ experiences of cultural stress. The combination of both theories provides a holistic perspective of how parental cultural stress impacts youth developmental outcomes, above and beyond normative family stressors.

## 10. Limitations and Future Directions

Though the current study sheds light on the importance of looking at multiple stressors at once, future research would benefit from further studying the interaction between acculturative and family stress and its cumulative impact on family and child functioning. It may be that these simultaneously affect mothers and fathers and are representative of greater variance in their reports of family cohesion and parenting behaviors. In the same vein, the current model could be expanded upon to include direct pathways from predictor variables to outcome variables, instead of just indirect associations, which was the focus of the current study. Lack of assessing fathers’ reports of general family stress at Time 1 was also a limitation, preventing the testing of unique and combined effects of both sources of stress on families from fathers’ perspectives. As mentioned previously, including the experiences of fathers in Latino families is vital given the centrality of fathers’ family role in this ethnic group. An additional limitation of the family stress measure is its low reliability. Future studies should replicate this type of analysis with a more reliable measure; however, the researchers kept the current instrument to highlight the importance of examining the compounded effect of normative stressors in addition to those related to acculturative stress. Further, a limitation of the current study includes the use of English competency pressures as the sole variable depicting the presence of acculturative stress. Though this was the only variable available in the current data, it has been shown to predict a large portion of acculturation stress-based experiences in immigrants’ lives, including posing barriers in communicating with peers and accessing institutions in the host country (Ali et al., 2024), as well as exposing immigrants to greater discrimination (Cheung et al., 2010). Future studies should examine these relationships in mothers and fathers utilizing a more comprehensive measure of acculturative stress, in addition to examining how the political landscape could inform reports of such stress. Finally, though the variables at hand had low to moderate correlations among one another, previous studies have suggested that small correlations can have practical utility, as supported by theoretical evidence for existing relationships (Rosenthal & Rubin, 1979). Further, the overall significance found in the structural models suggests that these are important pathways to consider when examining ways in which parents deal with and respond to acculturative and family stress.

## 11. Conclusions

Despite present limitations, the current study provides important contributions to the existing literature on acculturative stress. First, we demonstrate that acculturative stress manifests differently across parents, through gendered-specific reactions to stress in parenting. Second, we show evidence for elevating the role of family, as a cultural asset, for buffering the negative repercussions of cultural-specific stressors. Finally, we specify the need for paying attention to the ways in which acculturative stress, in addition to normative family stress, puts Latino immigrant families at a greater risk of experiencing family conflict and negative psychological health outcomes. These findings have implications for cultural stress and family stress research, and preventive interventions for families immersing themselves in a new cultural context.

## Figures and Tables

**Table 1:** Correlation Matrix, Means, and Standard Deviations for Study Variables (Mothers).

	1	2	3	4	5	6	7	8	9
1. Family Stress	----								
2. Acculturative Stress	−0.09	----							
3. Depression	0.12 **	0.28 **	----						
4. Family Cohesion	−0.04	−0.12 *	−0.30 **	----					
5. Warm Parenting (M)	0.05	0.05	−0.06	0.38 **	----				
6. Warm Parenting (C)	0.06	0.01	−0.05	0.17 **	0.18 **	----			
7. Harsh Parenting (M)	0.13 **	0.16 **	0.15 **	−0.15 **	−0.17 **	−0.08	----		
8. Harsh Parenting (C)	0.06	0.01	0.05	−0.11 *	−0.13 **	−0.23 **	0.21 **	----	
9. Internalizing Symptoms	−0.02	−0.08	0.02	−0.03	−0.07	−0.20 **	0.05	0.24 **	----
Mean	1.27	2.43	1.71	4.02	4.37	4.17	2.11	2.02	3.30
SD	1.20	1.19	0.48	0.52	0.55	0.74	0.62	0.70	2.68

Note: ** *p* < 0.01, * *p* < 0.05.

**Table 2:** Correlation Matrix, Means, and Standard Deviations for Study Variables (Fathers).

	1	2	3	4	5	6	7	8
1. Acculturative Stress	----							
2. Depression	0.27 **	----						
3. Family Cohesion	−0.15 **	−0.35 **	----					
4. Warm Parenting (F)	−0.10 **	−0.19 **	0.49 **	----				
5. Warm Parenting (C)	−0.08	−0.08	0.09	0.16 **	----			
6. Harsh Parenting (F)	0.21 **	0.22 **	−0.06	−0.12 *	−0.12 *	----		
7. Harsh Parenting (C)	0.04	0.07	−0.13 *	−0.11 *	−0.25 **	0.22 **	----	
8. Internalizing Symptoms	0.01	−0.02	−0.05	−0.09	−0.21 **	0.02	0.26 **	----
Mean	1.99	1.54	3.96	4.20	3.99	1.93	1.87	3.30
SD	0.95	0.43	0.52	0.55	0.93	0.61	0.72	2.68

Note: ** *p* < 0.01, * *p* < 0.05.

## Data Availability

The datasets presented in this article are not readily available because the data are part of an ongoing study. Requests to access the datasets should be directed to Nancy A. Gonzales.
